# Elevating the Optical Nonlinearity: Design, Synthesis, and Properties of a Mixed-Ligand Zinc(II) Metal–Organic Framework

**DOI:** 10.1021/acsami.4c22681

**Published:** 2025-03-05

**Authors:** Reza Abazari, Soheila Sanati, Marzieh Nadafan, David B. Cordes, Alexandra M. Z. Slawin, Alexander M. Kirillov

**Affiliations:** † Department of Inorganic Chemistry, Faculty of Science, 256565 University of Maragheh, P.O. Box Maragheh 55181-83111, Iran; ‡ Department of Physics, 121571 Shahid Rajaee Teacher Training University, P.O. Box Tehran 16788-15811, Iran; § School of Chemistry, 7486 University of St Andrews, North Haugh Fife, St Andrews KY16 9ST, U.K.; ∥ MINDlab: Molecular Design & Innovation Laboratory, Centro de Química Estrutural, Institute of Molecular Sciences, Departamento de Engenharia Química, Instituto Superior Técnico, 72971 Universidade de Lisboa, Av. Rovisco Pais, Lisbon 1049-001, Portugal

**Keywords:** coordination polymers, functional materials, nonlinear optical materials, two-photon absorption, third-harmonic generation

## Abstract

The molecular design of new compounds with enhanced third-order nonlinear optical (NLO) applications is an important research topic in the area of functional materials. The present study describes a new 3D metal–organic framework [Zn_3_(μ_3_-bta)_2_(μ_4_-ata)_2_]_n_ (abbreviated as NH_2_–Zn-MUM-6), generated solvothermally from a multicomponent system composed of zinc­(II) ions and 2-aminoterephthalic acid (H_2_ata) and 1H-benzotriazole (Hbta) as linkers. Apart from an intricate porous structure with a seh-4,6-*Imma* topology, this MOF features good thermal stability and exceptional NLO characteristics. These were investigated in detail by a Z-scan method, revealing the remarkably high values of third-order nonlinear refractive index (*n*_2_ = 30.83 × 10^–8^ cm^2^/W) and nonlinear absorption coefficient (β = 4.06 × 10^–4^ cm/W). An extended π-conjugation in both aromatic ligands, μ_3_-bta^–^ and μ_4_-ata^2–^, and their interaction with Zn centers contribute to the formation of a highly polarizable system, thus significantly enhancing its capacity for NLO behavior. This study broadens the family of nonlinear optical materials and provides insights into the relationship between the structure and properties of amino-functionalized mixed-linker MOFs from the viewpoint of optical applications such as all-optical switching and data transmission.

## Graphical abstract

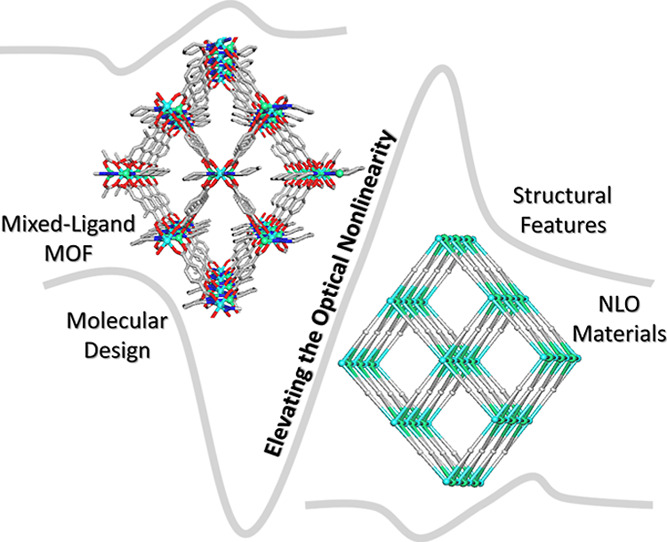

## 1 Introduction

The importance of third-order nonlinear optical (NLO) compounds in optical switching and limiting, laser protection, electro-optical signal processing, image transmission, environmental monitoring, and two-photon photodynamic therapy has enabled the design of unique NLO materials.^1^ These differ from organic and inorganic to hybrid materials with third-order NLO response, among which carbon nanodots, semiconductor quantum dots, conjugated organic molecules (porphyrins and phthalocyanines), polymers, as well as metal carbonyl clusters can be mentioned as examples.^2,3^ Inorganic solids are used in countless commercial NLO materials,^4,5^ although response time and processing potential represent the typical advantages of their organic counterparts.^6,7^ Greater flexibility and potential synergic effects of the hybrid materials with organic–inorganic components make them as outstanding candidates for practical applications.^8,9^ In particular, metal–organic frameworks (MOFs) can be considered as highly promising examples of such a type of hybrid materials for NLO applications.^10–12^

MOFs comprise metal ions or cluster nodes interconnected by organic linker ligands into porous frameworks.^13–15^ These materials possess a tremendous diversity of potential applications owing to their high porosity, chemical stability, and postsynthetic modifiability.^16–23^ NLO materials based on MOFs can also be designed and constructed in search for potential applications in optical limiting and as optical switches or mode-locked lasers.^24–26^ Different factors such as the metal ion type, interpenetration, guest species encapsulation, morphological characteristics, and even external stimuli like electric fields can affect the NLO behavior of MOFs.^27–30^ Besides, throughout the building block selection until the postmodification stage, various techniques are already available for adjusting the NLO performance in MOFs, which can be remarkably enhanced by the electron transfer between the organic ligands with conjugated π-bonding.^31–33^

In previous reports, the molecular design strategy for MOFs with excellent NLO performance was typically aimed at the modification of building blocks, such as extending the π-conjugated length of the ligands or introducing strong electron donor/acceptor groups and defect engineering.^34–36^ According to Cheng et al., the presence of variable valence electrons and vacant 3d orbitals represented the primary reasons behind the notable nonlinear optical behavior of Cu-MOFs, producing a conjugated structure via *d*-π interactions and consequently strengthening the NLO impact through p-electron delocalization.^37^ A study conducted by Shang et al. confirmed the significant effects of ligand-to-metal and metal-to-ligand charge transfers on the nonlinear response of MOFs.^38^ Based of the evidence presented by Zhang et al., the remarkable third-order NLO characteristics could be attributed to rich π-conjugation caused by the presence of pyridine and benzene moieties.^39^ Structural interpenetration in MOFs can also significantly impact their third-order NLO properties.^28^ In a recent study, Li at al. used a Z-scan method to inspect how the third-order NLO characteristics of a porphyrin MOF were influenced by the type of metal centers.^40^ Hou et al. reported that ultraviolet stimulation can enhance the third-order NLO performance of MOFs.^41^ In addition, we also showed that high-contrast nonlinear characteristics and optical switching can be influenced by the presence of free amino groups and open metal sites in MOFs.^42^

In fact, previous research revealed that the selection of structures containing electron-rich nitrogen sites in the linkers would effectively enhance the NLO function in MOFs.^5^ Thus, the linker selection contributes significantly to the development of NLO materials with distinctive optical characteristics, including an efficient exciton hopping, enhanced stimulated emission, or higher absorption efficiency.^12,25,43^ As a continuation of our general research line on the molecular design of new functional MOFs, in the present study we explored the solvothermal assembly in a multicomponent system composed of zinc­(II) ions, 2-aminoterephthalic acid (H_2_ata) and 1H-benzotriazole (Hbta). Hence, we report herein the design, synthesis, full characterization, and detailed investigation of nonlinear optical properties of a new 3D MOF [Zn_3_(μ_3_-bta)_2_(μ_4_-ata)_2_]_*n*_, abbreviated as NH_2_–Zn-MUM-6. Apart from an intricate porous structure with a seh-4,6-*Imma* topology, this compound features exceptional NLO characteristics. This research thus contributes to widening the family of nonlinear optical materials and provides deeper insights into the relationship between structure and properties of amino-functionalized mixed-linker MOFs from the viewpoint of optical applications.

## 2 Experimental Section

### 2.1 Preparation of [Zn_3_(μ_3_-bta)_2_(μ_4_-ata)_2_]_*n*_ (NH_2_–Zn-MUM-6)

A solvothermal crystallization technique was employed to synthesize NH_2_–Zn-MUM-6. A mixture of Zn­(NO_3_)_2_·6H_2_O (0.36 mmol, 0.11 g), 2-aminoterephthalic acid (H_2_ata; 0.24 mmol, 0.043 g), 1H-benzotriazole (Hbta; 0.24 mmol, 0.028 g), and *N*,*N*-dimethylformamide (DMF, 7 mL) was added to a 12 mL Teflon-lined autoclave. This was sealed and kept at 80 °C for 60 h, followed by gradual cooling (6 °C/h) and opening the autoclave. The obtained orange-brown crystals (Figure S1, Supporting Information) were washed with DMF and ethanol, and dried at 110 °C for 18 h, resulting in a 71% yield of NH_2_–Zn-MUM-6 (based on H_2_ata). d.p. > 330 °C. IR data (KBr pellet, cm^–1^): 556­(m), 647­(m), 748­(vs), 751­(vs), 859­(w), 934­(w), 1207(s), 1262­(m), 1380­(m), 1449­(m), 1537­(w), 1565­(m), 1610­(w), 1669­(w), 2950­(w), 2965­(w), 3335­(w), and 3489­(w).

### 2.2 Nonlinear Optical Assessment

A homemade Z-scan instrument was utilized to study the NLO profile of NH_2_–Zn-MUM-6 at 532 nm, with a laser having the 190 mm focal length. The laser was focused on the samples and the beam waist radius, ω_0_, varied from 32 to 45 μm at the beam focus. A translation stage with high accuracy was utilized for sample fixation, with 0.2 mm movements in each step. The intensity of transmitted light was examined by a Silicon PIN Photodiode detector.

## 3 Results and Discussion

### 3.1 Structural Features of NH_2_–Zn-MUM-6

The structure of [Zn_3_(μ_3_-bta)_2_(μ_4_-ata)_2_]_*n*_ (NH_2_–Zn-MUM-6) reveals a 3D metal–organic framework (Figure 1) that is assembled from the two types of zinc­(II) centers (Zn1, Zn2) and two types of linkers, namely μ_4_-ata^2–^ and μ_3_-bta^–^. The Zn1 centers are six-coordinate and exhibit an ideal {ZnN_2_O_4_} octahedral environment, which is occupied by two N donors from two μ_3_-bta^–^ and four O donors from four μ_4_-ata^2–^ linkers. The Zn2 centers are four-coordinate in a distorted {ZnN_2_O_2_} tetrahedral fashion, wherein the coordination geometry is filled by a pair of N atoms from two μ_3_-bta^–^, and two O atoms from two μ_4_-ata^2–^ moieties (Figure 1a). To get further insight into the resulting 3D framework (Figure 1b), it was simplified from a topological perspective following the concept of an underlying net and using ToposPro software.^44–47^ The simplification considered both the {Zn_2_(bta)_2_}^2+^ motifs (based on the Zn_2_ centers) and the Zn_1_ centers as the 6-connected and topologically equivalent nodes which, along with the 4-connected μ_4_-ata^2–^ nodes, form a 3D metal–organic framework (Figures 1c and S3). It can be classified as a binodal 4,6-linked net with a seh-4,6-*Imma* topology and a point symbol of (3^2^.6^2^.7^2^)­(3.^4^4^2^.6^4^.7^5^). Herein, the (3^2^.6^2^.7^2^) and (3^4^.4^2^.6^4^.7^5^) notations correspond to the μ_4_-ata^2–^ and {Zn_2_(bta)_2_}^2+^/Zn1 nodes, respectively. An interesting feature of the NH_2_–Zn-MUM-6 consists in the presence of large voids (Figure S4, Supporting Information), which occupy up to 37% of the unit cell volume according to the analysis performed with Mercury software.

**Figure 1:**
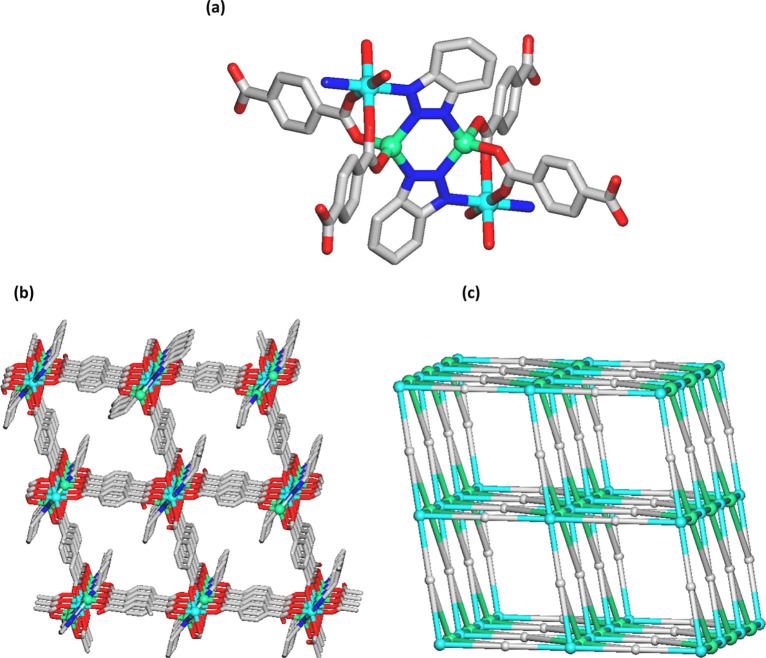
Crystal structure of NH_2_–Zn-MUM-6. (a) Coordination environments around Zn1 (cyan) and Zn2 (green) centers and connectivity of ligands; C gray, O red, N blue. (b) 3D MOF along the *c* axis and its (c) topological representation showing a dinodal 4,6-connected net with a seh-4,6-*Imma* topology; centroids of 6-linked {Zn_2_(bta)_2_}^2+^ nodes (green balls), 6-linked Zn1 atoms (cyan balls), centroids of 4-linked μ_4_-ata^2–^ nodes (gray). In (a,b), H atoms and disordered NH_2_ groups in μ_4_-ata^2–^ are not shown for clarity.

### 3.2 Characterization of NH_2_–Zn-MUM-6

To remove residual solvent from the voids of the MOF and perform the activation of the sample, it was kept in anhydrous ethanol for 2 days for solvent exchange. Then, the NH_2_–Zn-MUM-6 sample was dried under vacuum at ambient temperature for 48 h, followed by heating at 140 °C for 24 h to complete the activation. The activated NH_2_–Zn-MUM-6 was used in further analyses, including the N_2_ sorption studies and optical properties investigation. PXRD analysis was conducted to confirm a good phase purity and crystallinity of NH_2_–Zn-MUM-6, as attested by similarity of experimental and simulated patterns (Figure 2a).

**Figure 2:**
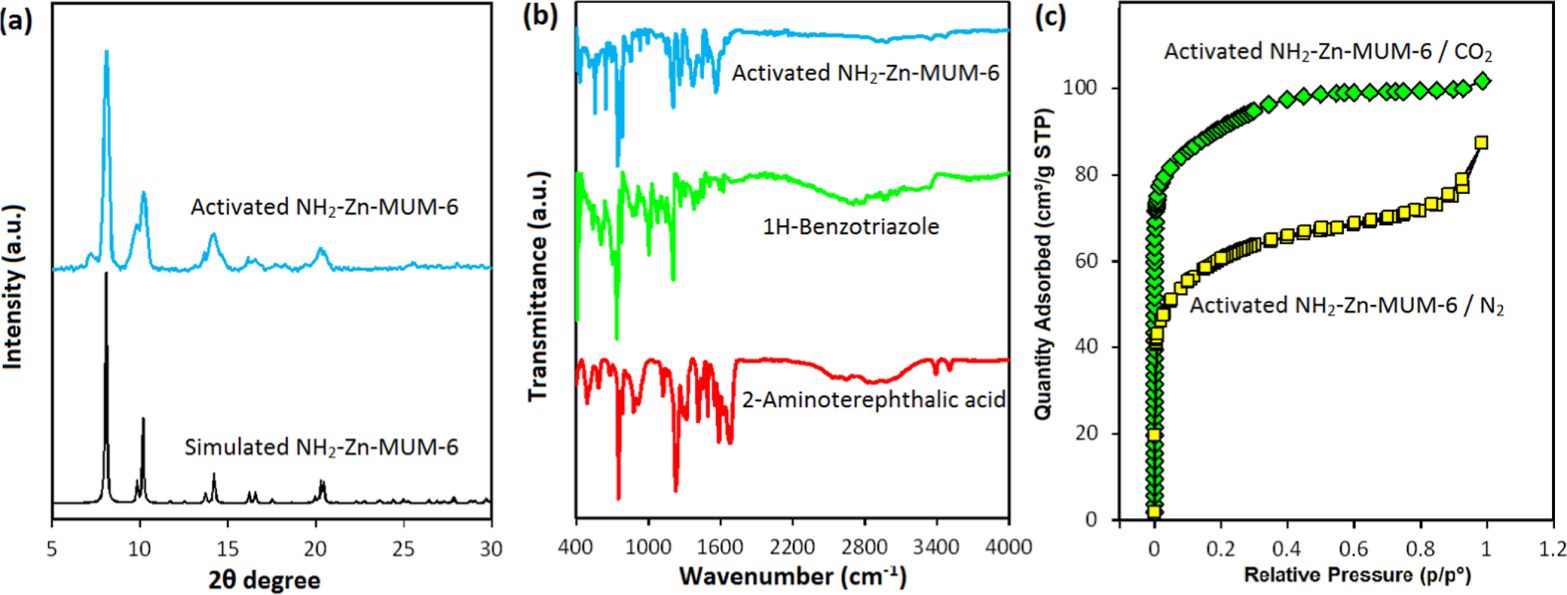
PXRD patterns (a), FT-IR spectra (b), and CO_2_ and N_2_ adsorption–desorption isotherms (c) of studied materials.

The FTIR spectrum of NH_2_–Zn-MUM-6 (Figure 2b) reveals highly intense and broad bands with maxima at 1662 and 1403 cm^–1^, attributed to the ν_as_ and ν_s_ vibrations of the COO^–^ groups within μ_4_-ata^2–^ ligands.^26,42,48^ The bands with maxima at 3390 and 3451 cm^–1^ in NH_2_–Zn-MUM-6 associate with ν­(NH) of primary amine functional groups.^26,42,49^ The moderate intensity bands at 758 and 1096 cm^–1^ are due to the Zn–O stretching and C–O bending vibrations, respectively.^50^ There is also a number of typical bands arising from the presence of μ_3_-bta^–^ ligands.^51,52^ Figure 2c highlights the microporosity of NH_2_–Zn-MUM-6 considering the reversible type-I isotherm and the steep N_2_ uptake under low-pressure conditions (*P*/*P*_0_ < 0.07) in this material.^26,53^ The surface area (BET) of 117 m^2^ g^–1^ was determined for NH_2_–Zn-MUM-6, which also exhibits a 185 cm^3^ g^–1^ CO_2_ uptake at *P/P*_0_ = 0.2. Moreover, higher CO_2_ adsorption temperatures and some MOF-CO_2_ interactions can be used to overcome the energy barrier for gas diffusion through the NH_2_–Zn-MUM-6 pores. The microporosity of NH_2_–Zn-MUM-6 is highlighted by the typical type-I adsorption isotherms of CO_2_. The total pore volume of 0.13 cm^3^ g^–1^ can be achieved considering the empirical CO_2_ data at *P/P*_0_ = 0.2. The thermogravimetric analysis of NH_2_–Zn-MUM-6 was conducted under airflow, indicating the stability of desolvated metal–organic framework up to ∼300 °C (Figure S5, Supporting Information). The obtained MOF also reveals a good shelf life stability in the solid state. The band gap evaluation was performed demonstrating a significant UV light absorbance by NH_2_–Zn-MUM-6 (Figure S6, Supporting Information). The band gap, 2.95 eV, was estimated by the following formula:^26,54^ α*h*υ = *A*(*h*υ-*E*_g_)^*n*/2^, with α, *h*, υ, *A*, *E*_g_, and *n* referring to absorption coefficient, Planck’s constant, incident light frequency, constant, band gap energy, and optical transition properties. SEM analysis of NH_2_–Zn-MUM-6 (Figure S2a, Supporting Information) shows that this material has irregular polyhedra. The TEM analysis of the sample after being dispersed for 30 min under ultrasonic treatment reveals particles with a size of about 70 nm (Figure S2b, Supporting Information).

### 3.3 Nonlinear Optical Behavior

The NLO behavior of the obtained MOF was studied in the solid state using a Z-scan technique.^55^ The third-order NLO characteristics, including nonlinear absorption (NLA) and nonlinear refraction (NLR), are determined by the open and closed apertures (referred to as OA and CA, respectively) of Z-scan. The intensities associated with the transmitted or the input laser powers are recorded by two detectors. A typical Z-scan experimental setup scheme is shown in Figure S7.^56,57^ The eq 1 was used to calculate the NLA coefficient, β, estimated by the OA Z-scan (eq 1)­

1 $$\beta =\frac{2\sqrt{2}\left(1-T\left(z\right)\right)}{I_{0}L_{eff}}\left(1+\frac{z^{2}}{z_{0}^{2}}\right),in,which,\left\{\begin{array}{l} L_{eff}=\left[\left(1-\exp\left(-\alpha L\right)\right)/\alpha \right]\\ z_{0}=\pi \omega_{0}^{2}/\lambda \\ \alpha =-\ln\left(-P/P_{0}\right)/L \end{array}\right\}$$

Here, *L*_eff_,*I*_0_ α, *z*_0_, *P*_0_, and *P* show the respective symbols for the effective thickness, laser intensity, linear absorption, Rayleigh length, and the input and the output powers. Also, *T*(*z*) presents the normalized transmittance assuming *z* = 0. According to the data presented in Figure 3, the 2-photon absorption (TPA) effects for different *z* positions resulted from the minimum beam energy density at the focus. The sample’s NLA coefficient shows an increment as the incident power of laser increases. The OA experimental data were employed for calculating the NLA coefficient (β). The eqs 2 and ([Disp-formula eq3]) were utilized to confirm the NLA-related experimental and theoretical results.

2 $$T_{norm}\left(z\right)=\sum_{m=0}^{\infty }\frac{{\left[-q_{0}\left(z,0\right)\right]}^{m}}{{\left(m+1\right)}^{3/2}}$$

3 $$q_{0}\left(z,t\right)=\left(\beta I_{0}L_{eff}\right)/\left(1+z^{2}/z_{0}^{2}\right)$$

**Figure 3:**
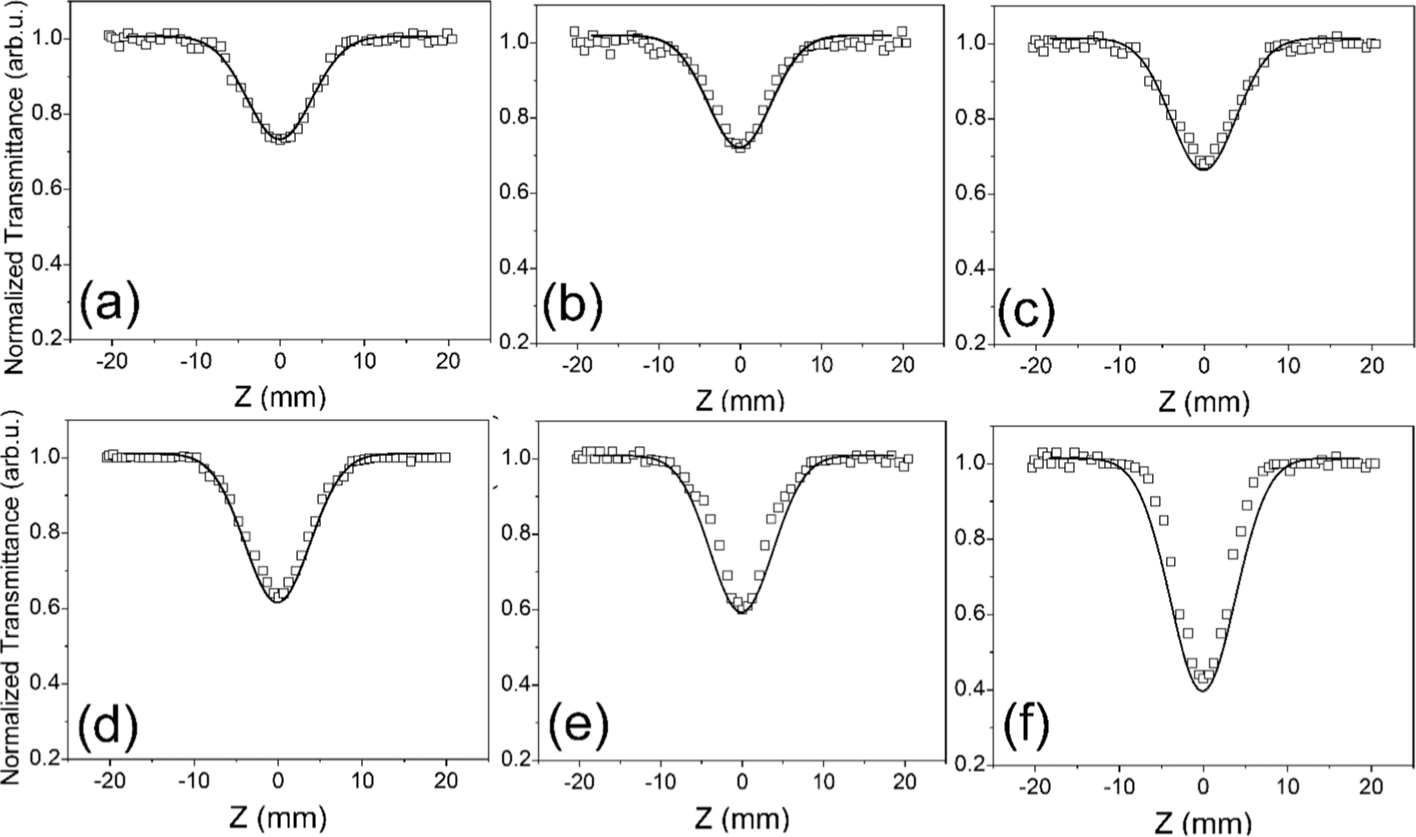
Normalized OA Z-scan profile of NH_2_–Zn-MUM-6 at (a) 10, (b) 20, (c) 30, (d) 40, (e) 50, and (f) 60 mW of laser power. The solid lines represent the theoretical curves, while the squares represent the experimental points.

The data obtained by the CA Z-scan revealed the peak–valley or valley–peak arrangements for transmittance, suggesting negative/positive NLR indices and respective self-defocusing or focusing patterns for the samples. As revealed by Figure 4, an increase in the incident power of the laser led to a growth of the positive nonlinear refraction characteristics. The relation between the differences in peak and valley transmittance, Δ*T*_*P*–*V*_, and the on-axis shift for the phase Δφ_0_ are expressed by eq 4

4 ΔTP−V=0.406(1−S)0.25|Δφ0|Δφ0=(2π/λ)n2I0Leff

Here, the NLR index is indicated by *n*_2_. Besides, the following eq 5 expresses the fitted graph with experimental values of CA^58,59^

5 $$T_{norm}=1-\frac{4x\Delta \varphi_{0}}{\left(1+x^{2}\right)\left(9+x^{2}\right)},where,x=\frac{z}{z_{0}}$$

**Figure 4:**
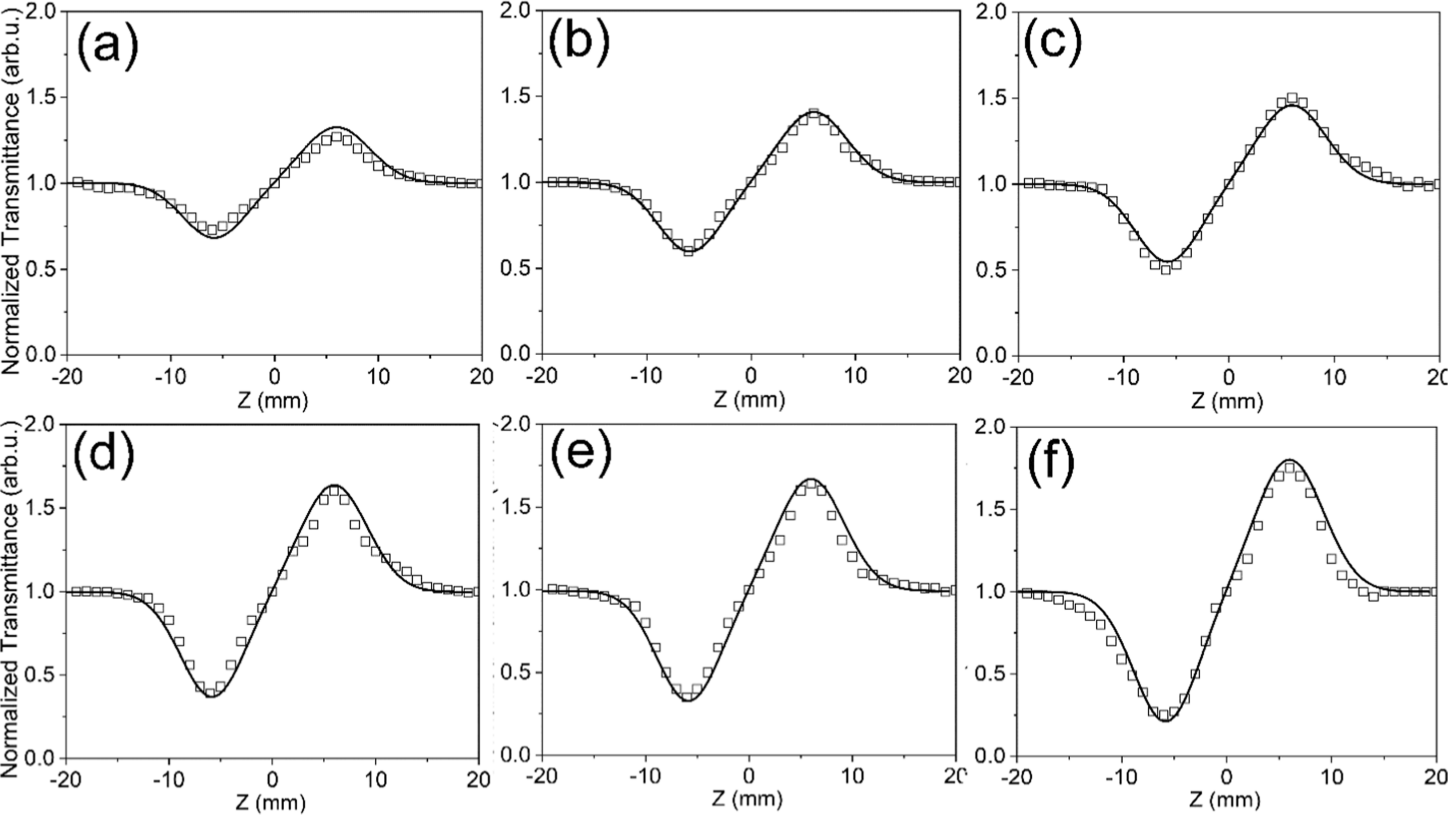
Normalized CA Z-scan curves of NH_2_–Zn-MUM-6 at (a) 10, (b) 20, (c) 30, (d) 40, (e) 50, and (f) 60 mW of laser power. The solid lines represent the theoretical curves, while the squares represent the experimental points.

The NLO susceptibility of samples was evaluated by the following eqs 6 and ([Disp-formula eq7])^55^

6 $$Re\left(\chi^{\left(3\right)}\right)\left(esu\right)=\frac{10^{-4}}{\pi }\varepsilon_{0}c^{2}n_{0}^{2}n_{2}\left(\frac{cm^{2}}{W}\right)$$

7 $$Im\left(\chi^{\left(3\right)}\right)\left(esu\right)=\frac{10^{-2}}{4\pi^{2}}\varepsilon_{0}c^{2}n_{0}^{2}\lambda \beta \left(\frac{cm}{W}\right)$$

From the viewpoint of light–matter interaction, susceptibility is a concept indicating how a material can respond to applied electric fields. It is also responsible for quantifying the material’s polarization, representing electric field exposure conditions under which the induced separation of positive/negative charges occurs.

Table 1 shows the NLR index (*n*_2_) and the NLA coefficient (β) of NH_2_–Zn-MUM-6, with the respective values in the range of (8.66–30.83) × 10^–8^ cm^2^/W and (5.38–12.62) × 10^–3^ cm/W. These values at various incident powers of laser are summarized in Figure 5.

**Table 1:** Nonlinear Optical Properties of NH_2_–Zn-MUM-6

α(**cm** ^–1^)	*P*(mW)	***L***_**eff**_(**mm**)	n2×10−8(cm2/W)	β×10−3(cm/W)	Re|χ(3)|×10−5(esu)	Im|χ(3)|×10−4(esu)	|χ(3)|×10−4(esu)
2.30	10	0.89	8.66	5.38	3.42	1.71	1.74
2.27	20	0.89	15.48	5.83	4.45	1.85	1.90
2.17	30	0.89	20.15	6.03	4.85	1.91	1.97
2.28	40	0.89	21.95	8.06	4.99	2.55	2.60
2.15	50	0.89	22.69	8.12	1.91	2.57	2.58
2.56	60	0.88	30.83	12.62	6.81	4.01	4.06

**Figure 5:**
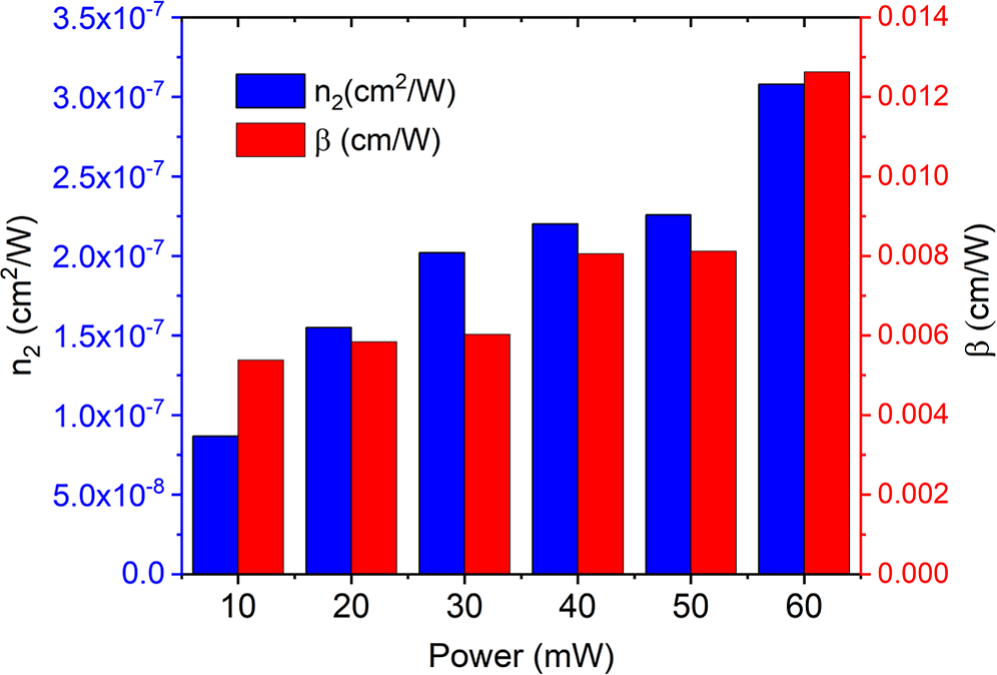
NLR index (left or blue bars) and NLA coefficient (right or red bars) of NH_2_–Zn-MUM-6 at 10, 20, 30, 40, 50, and 60 mW of laser power.

Nonlinearity can arise from various factors such as electronic, molecular, electrostrictive, or thermal effects. In the latter case, thermal influences primarily contribute to the nonlinearity, with relaxation times in the millisecond range and peak-to-valley differences exceeding 1.7 *Z*_R_. Nonlinear materials like NH_2_–Zn-MUM-6 often show a significant third-order susceptibility (χ^(3)^), which is responsible for effects such as the nonlinear refractive index and third-harmonic generation. The electronic transitions within the material can be highly responsive to the electric field of the incident light. NH_2_–Zn-MUM-6 may exhibit TPA, where a simultaneous absorption of two photons leads to an excited state. The phenomenon of TPA occurs when the energy of the laser surpasses half of the material’s band gap (*h*ν > *E*_g_/2).

An increase in laser power enhances the NLO effects in NH_2_–Zn-MUM-6. The polarity of this MOF in terms of its NLO properties becomes more significant at higher laser powers. This does not refer to the intrinsic molecular polarity but rather to the material’s response to the incident light intensity in a nonlinear manner. There are some factors related to the nonlinear optical behavior of NH_2_–Zn-MUM-6, the structure of which consists of zinc ions coordinated by two types of organic ligands. The extended conjugation in these aromatic ligands and their interaction with metal centers can create a highly polarizable system, prone to exhibit NLO effects. On the other hand, π-conjugated systems within the organic ligands permit significant electron delocalization, which enhances the nonlinear optical response. The combination of metal centers and organic linkers in NH_2_–Zn-MUM-6 also enhances the overall polarizability of the material. When subjected to intense light, the induced polarization can lead to the NLO phenomena such as self-focusing, self-defocusing, and/or nonlinear absorption. NH_2_–Zn-MUM-6 can also show some network flexibility in response to external field. This flexibility can contribute to the NLO properties by allowing the material to dynamically adjust its electronic and structural configuration under intense light. Polarizable valence electrons in the zinc ions can interfere with the π-electron conjugation system, resulting in recombination or distortion of the electron cloud. This interference modifies the NLO properties of MOFs.^36^

A potential role of MOFs in nonlinear optics has increasingly stimulated the research in this area.^60–63^ A comparison of the NH_2_–Zn-MUM-6 characteristics with previously reported data on related materials is presented in Table S2. For example, Chen et al. demonstrated that the distinct RSA responses of cage-supported cluster-organic frameworks primarily arise from the inclusion of clusters and the abundant π···π interactions within their structures.^64^ In a related study, Cheng et al. highlighted that the NLO performances of copper­(I) sulfide clusters depend significantly on the structural variations of the cluster core, surpassing those of metal-chalcogenide materials.^65^ Gu et al. reported that the surface anchored metal–organic frameworks (SURMOFs) exhibit a surface-enhanced second harmonic generation effect, attributed to the electronic transition from the S0 to S2 states.^66^ Moreover, a dense incorporation of porphyrin molecules into the framework, alongside an extensive π-electron delocalization, is responsible for the exceptionally strong NLO absorption observed in SURMOFs.^67^

Li and colleagues confirmed that the interpenetration of porphyrinic groups within the framework enhances electron delocalization and facilitates optical limiting (OL) performance.^28^ Rath et al. investigated the TPA-induced photoluminescence of Cd­(II) MOFs incorporating acceptor-π-donor-π-acceptor linkers.^34^ They found that, compared to a Zn­(II) MOF, some Cd­(II)-based MOFs exhibit an enhanced TPA. Different properties were attributed to factors such as chromophore density, interpenetration degree, chromophore orientation, and π···π interactions between the network components, all influencing the NLO behavior.^34^ The development of the d−π conjugation effects and further regulation of defects in TCPP/UiO-66 resulted from introducing nitrogen-rich sites with the ability of metal ion accommodation in TCPP (tetrakis­(4-carboxyphenyl)­porphyrin) rings, including Co­(II), Ni­(II), Cu­(II), and Zn­(II).^36^ An important role of TCPP consists in considerable electron delocalization enhancement and the effective electronic structure modulation by defects.^36^ A photoresponsive Zn­(II) MOF was used by Yu et al. as a precursor to synthesize bimetallic MOFs (ZnCu-MOF and ZnCd-MOF) by metal exchange,^41^ highlighting the contribution of metal ions with various electron configurations to the improvement of NLO characteristics. Under these conditions, electron transfer is promoted due bandgap adjustment and electron delocalization enhancement in MOFs.^41^ Furthermore, the doping of Co, Zn, and Ni into MOFs increases the selectivity of electronic transitions, resulting in an enhanced energy barrier required for these transitions.^67^ An extended conjugation of organic ligands and their interaction with metal ions may create a highly polarizable system, enhancing its potential for NLO behavior.^68^ Furthermore, the π-conjugated systems within aromatic ligands facilitate electron delocalization, further amplifying the NLO response. This behavior contributes to remarkable NLO properties of NH_2_–Zn-MUM-6.

## 4 Conclusion

In summary, a new zinc­(II) 3D MOF, [Zn_3_(μ_3_-bta)_2_(μ_4_-ata)_2_]_*n*_ (NH_2_–Zn-MUM-6), with a seh-4,6-*Imma* topology was assembled from two types of ligands and fully characterized in the present work. The Z-scan technique was employed to examine its nonlinear optical behavior in the solid state at different laser intensities. The remarkably high values of third-order nonlinear refractive index (*n*_2_ = 30.83 × 10^–8^ cm^2^/W) and nonlinear absorption coefficient (β = 4.06 × 10^–4^ cm/W) were revealed by NH_2_–Zn-MUM-6. An extended π-conjugation in both aromatic ligands, μ_3_-bta^–^ and μ_4_-ata^2–^, and their interaction with Zn centers contributes to the formation of a highly polarizable system, thus significantly enhancing its capacity for NLO behavior. Additionally, the π-conjugated ligands promote extensive electron delocalization, further intensifying the material’s NLO response. Influenced by lone electron pairs in their atoms, nitrogen centers in the ligands contribute to the generation of donor–acceptor (D–A) interactions due to intramolecular charge transfer. This study extends the growing family of MOFs with the NLO properties to an additional example with excellent nonlinear optical performance. Further research aiming at the molecular design and synthesis of related metal–organic frameworks and tuning the properties of NH_2_–Zn-MUM-6 by postsynthetic modification and/or composition with other materials can be envisaged. These research lines are currently being explored in our research laboratories.

## Supplementary Material





## Notes

Materials and general methods, crystallographic data, additional characterization and comparison (Figures S1–S7 and Tables S1 and S2) (PDF)
crystallographic data in CIF format (CCDC 2365799) and supporting literature (CIF)
