# Supplementary material for: Elevating the Optical Nonlinearity: Design, Synthesis, and Properties of a Mixed-Ligand Zinc(II) Metal–Organic Framework
Source: ACS Appl Mater Interfaces. 2025 Mar 5;17(11):17066–74. doi: 10.1021/acsami.4c22681 (PMC12818725; doi:10.1021/acsami.4c22681)
Supplement: Supplementary file 1 [file am4c22681_si_001.pdf]

Supporting Information (SI)

Elevating the Optical Nonlinearity: Design, Synthesis, and Properties of a Mixed-Ligand Zinc(II) Metal–Organic Framework

Reza Abazari,[†,*] Soheila Sanati,[†] Marzieh Nadafan,[‡] David B. Cordes,[§] Alexandra M. Z. Slawin,[§] Alexander M. Kirillov[#,*]

[†]*Department of Inorganic Chemistry, Faculty of Science, University of Maragheh, P.O. Box 55181-83111, Maragheh, Iran*

[‡]*Department of Physics, Shahid Rajaee Teacher Training University, P. O. Box 16788-15811, Tehran, Iran*

[§]*School of Chemistry, University of St Andrews, North Haugh, St Andrews, Fife KY16 9ST, United Kingdom*

[#]*MINDlab: Molecular Design & Innovation Laboratory, Centro de Química Estrutural, Institute of Molecular Sciences, Departamento de Engenharia Química, Instituto Superior Técnico, Universidade de Lisboa, Av. Rovisco Pais, 1049-001, Lisbon, Portugal*

E-mails: reza.abazari@maragheh.ac.ir (R. Abazari); kirillov@tecnico.ulisboa.pt (A.M. Kirillov).

Supporting Information Contains: Materials and general methods, crystallographic data, additional characterization and comparison (Figures S1-S6 and Tables S1-S2), crystallographic data in CIF format (CCDC 2365799) and supporting literature (PDF).

Materials. All reagents and solvents (analytical grade) were used as received from commercial sources: $Zn(NO_3)_2{\cdot}6H_2O$ (Merck, 98%), 2-aminoterephthalic acid (Aldrich, 96%), 1H-benzotriazole (Merck, 98%), ethanol (EtOH), and N,N-dimethylformamide (DMF, Merck).

General Methods. The N_2 adsorption/desorption isotherms were measured on an activated sample at liquid nitrogen temperature (77 K) by using a Micromeritics ASAP 2020 analyzer. The specific surface area was calculated by the Brunauer-Emmett-Teller (BET) method. A Thermo Nicolet IR 100 FTIR instrument was employed to record the FTIR spectra. The Powder X-ray diffraction (PXRD) patterns of the samples were recorded on a Philips X'pert diffractometer equipped with a Cu Kα radiation source. Morphological features were studied using a Transmission Electron Microscope (TEM) from JEOL Japan, model JEM-2100. The SEM images were obtained on a Hitachi S-1460 instrument with 15 kV accelerating voltage. Prior to the SEM examination, the samples were treated with ethanol, dried on a silica wafer, and sputter-coated by gold. Thermogravimetric analysis (TGA) data were acquired on a Shimadzu DTG-60H instrument from 30 to 800 °C at a heating rate of 10 ℃ min^{-1} under N_2 gas flow.

Single Crystal X-ray Diffraction. X-ray diffraction data for NH_2-Zn-MUM-6 were collected at 173 K using a Rigaku SCXmini CCD diffractometer with a SHINE monochromator [Mo Kα radiation (λ = 0.71073 Å)]. Intensity data were collected using ω steps accumulating area detector images spanning at least a hemisphere of reciprocal space. Data were collected and processed (including correction for Lorentz, polarization and absorption) using CrystalClear.[S1] The structure was solved by dual-space methods (SHELXT)[S2] and refined by full-matrix least-squares against F^2 (SHELXL-2019/3).[S3] Non-hydrogen atoms were refined anisotropically, and hydrogen atoms were refined using a riding model. The structure showed disorder in the orientation of the benzotriazolate ligand, which was modelled in two orientations with restraints to bond distances and thermal motion. This disorder was complicated by one of the modelled positions lying in a crystallographic mirror plane. The aminoterephthlate ligand showed some elongated thermal ellipsoids, particularly for the amine group, suggesting the possibility of further disorder here; however, this could not be successfully modelled. The structure showed a high proportion

of void space (836 Å^3, 37% of unit cell volume) and the SQUEEZE[S4] routine implemented in PLATON[S5] was used to remove the contribution to the diffraction pattern of the unordered electron density in the void spaces. All calculations except SQUEEZE were performed using the Olex2 interface.[S6] Selected crystallographic data are presented in Table S1. CCDC 2365799 contains the supplementary crystallographic data for this paper. These data can be obtained free of charge from The Cambridge Crystallographic Data Centre via www.ccdc.cam.ac.uk/structures.

Topological Analyses. Topological analysis of NH_2-Zn-MUM-6 was performed with ToposPro, following the concept of simplified network.[S7-S10] For this purpose, the bridging ligands were reduced to their centroids, while their connectivity to zinc(II) centers was preserved.

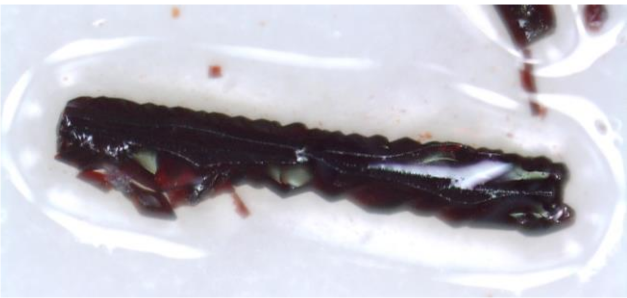

Figure S1. Light microscope image of solvated bulk NH_2-Zn-MUM-6 crystals.

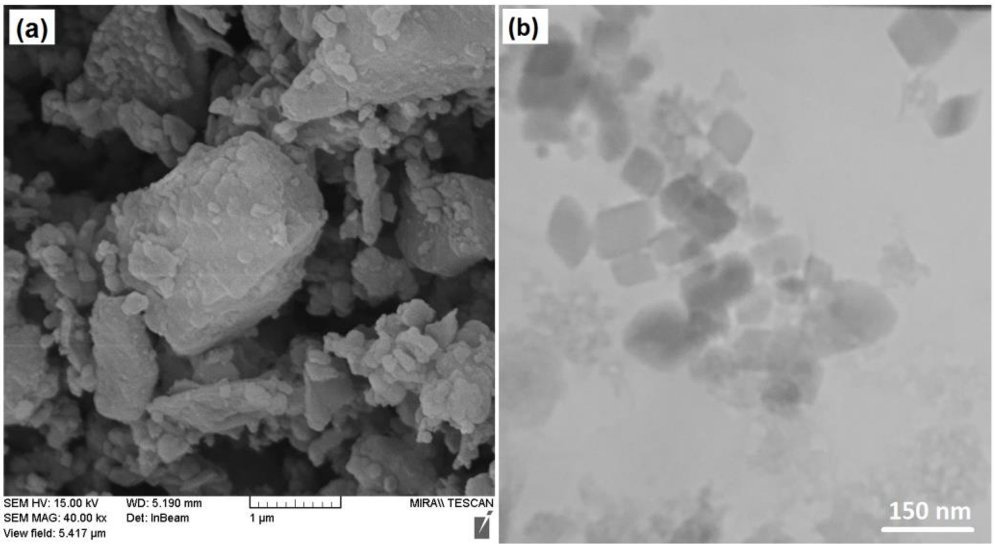

Figure S2. SEM (a) and TEM (b) images of NH_2-Zn-MUM-6.

(a) (b)

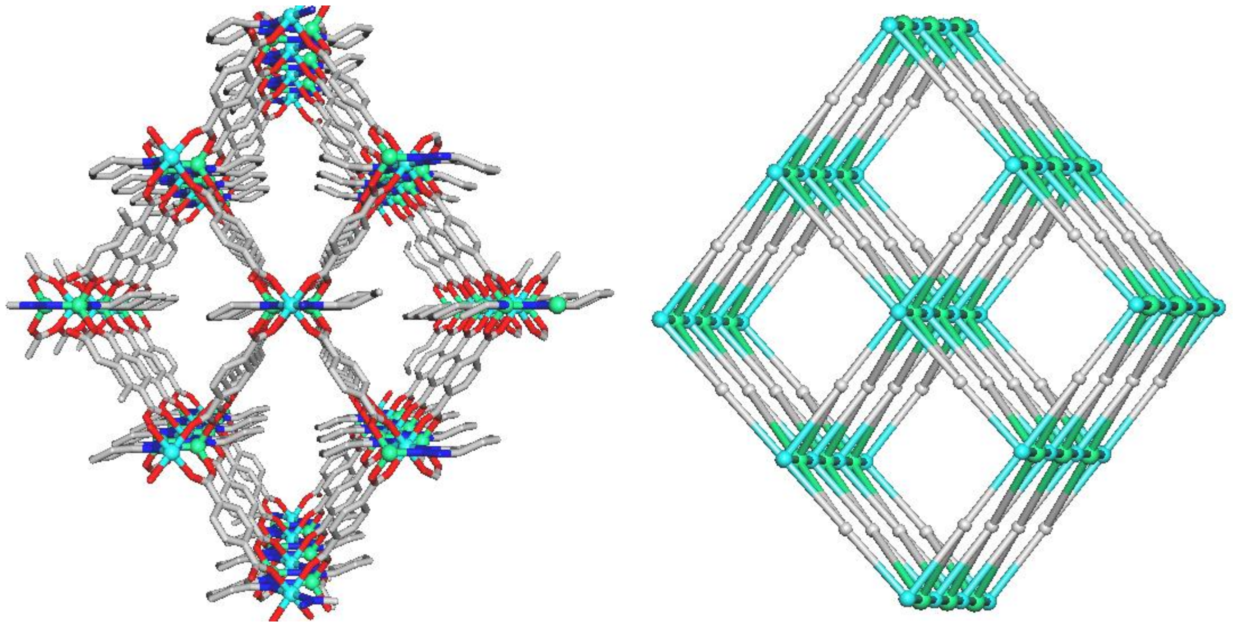

Figure S3. Perspective views of the crystal structure of NH_2-Zn-MUM-6 (a) and its topological representation (b). Views are along the *c* axis. In (a), color codes are: Zn1 cyan, Zn2 green, C gray, O red, N blue; H atoms and disordered NH_2 groups in μ_4-ata^{2-} are not shown for clarity. In (b), centroids of 6-linked $\{Zn_2(bta)_2\}^{2+}$ nodes (green balls), 6-linked Zn1 atoms (cyan balls), centroids of 4-linked μ_4-ata^{2-} nodes (gray).

(a) (b)

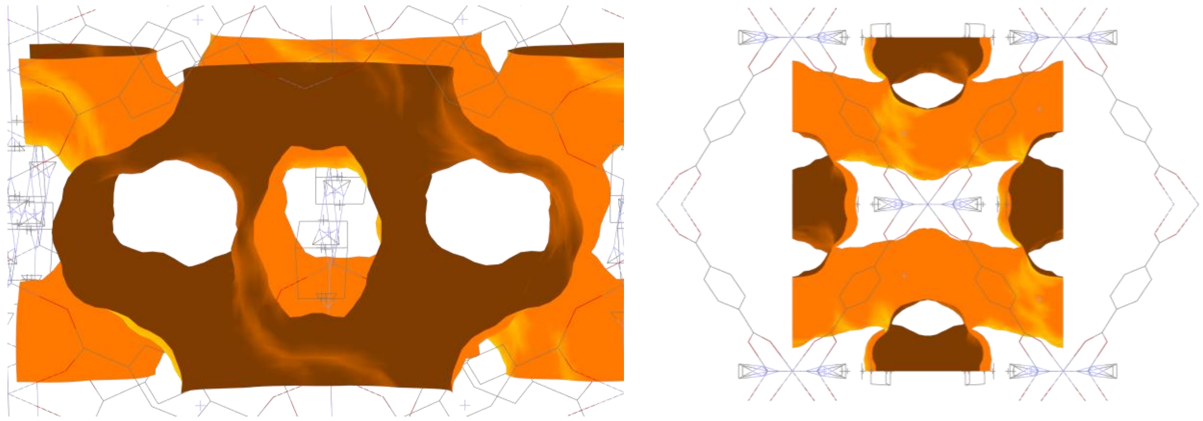

Figure S4. Representation of voids (orange surfaces, calculated using Mercury software) in the crystal structure of NH_2-Zn-MUM-6; views along the *a* (a) and *c* (b) axis. A wireframe representation of 3D metal-organic network is used.

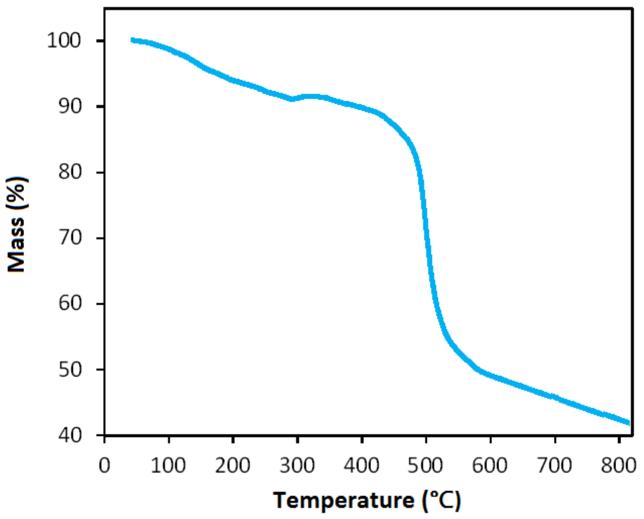

Figure S5. Thermogravimetric analysis of solvated bulk NH_2-Zn-MUM-6 crystals synthesized via hydrothermal method. Thermal effect in the ~60-250 °C range refers to the elimination of solvent molecules; the desolvated MOF structure is stable up to ~300 °C.

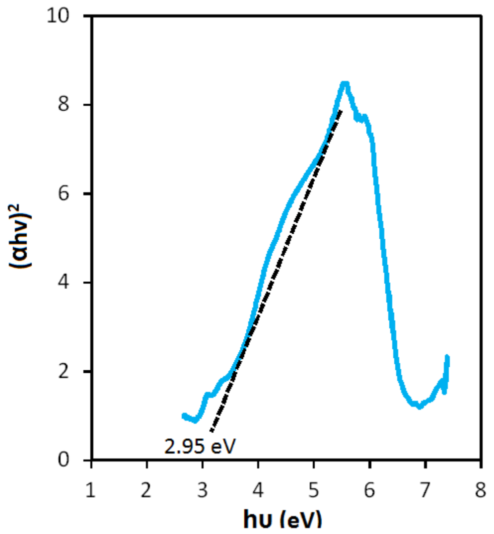

Figure S6. Band gap evaluation of NH_2-Zn-MUM-6 from the plot of $(\alpha h\nu)^2$ vs. the absorbed photon energy (eV).

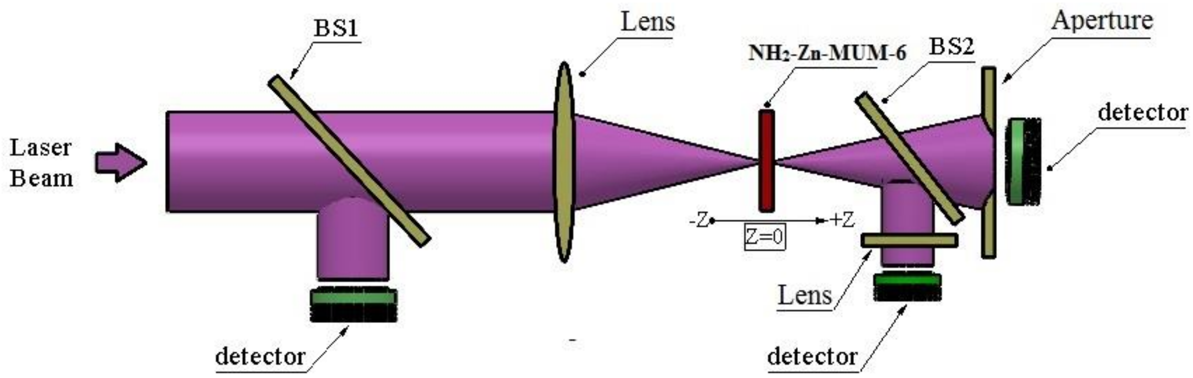

Figure S7. Experimental Z-scan setup.

Table S1. Selected Crystallographic Data for NH_2-Zn-MUM-6.

	NH_2-Zn-MUM-6
formula	$C_{28}H_{18}N_8O_8Zn_3$
fw	790.61
crystal description	Orange prism
crystal size [mm^3]	0.42×0.31×0.19
temperature [K]	173
space group	*C*2/*m*
a [Å]	14.3931(7)
b [Å]	17.3444(9)
c [Å]	9.1589(5)
β [°]	101.817(5)
vol [Å]^3	2238.0(2)
Z	2
ρ (calc) [g/cm^3]	1.173
μ [mm^{-1}]	1.638
F(000)	792
reflections collected	13705
independent reflections (R_{int})	3746 (0.0232)
parameters, restraints	193, 295
GoF on F^2	1.032
R_1 [$I > 2\sigma(I)$]	0.0355
wR_2 (all data)	0.1221
largest diff. peak/hole [e/Å^3]	0.681, -0.393

Table S2. Nonlinear Optical Properties of NH_2-Zn-MUM-6 in Comparison with Related Materials.

Materials	*λ (nm)*	$n_2 \times 10^{-8} (cm^2/W)$	*Self-defoc./ Self-foc.*	$\beta \times 10^{-3} (cm/W)$	*TPA/SA/RSA*	*Ref.*
Cu_6[1]	532	---	---	1×10^{-5}	RSA	[S11]
Cu_8[2]	532	---	---	3.5×10^{-5}	RSA	[S11]
SURMOFs[3]	400	---	---	9.29×10^{-1}	SA	[S12]
SURMOFs	800	---	---	1×10^{-4}	RSA	[S12]
BIF-134[4]	532	---	---	3.6×10^{-5}	RSA	[S13]
BIF-135[5]	532	---	---	6.9×10^{-4}	RSA	[S13]
$[Zn_2(TPyP)(AC)_2]$[6]	532	---	---	3.61	RSA	[S14]
$[Zn_3(TPyP)(H_2O)_2(C_2O_4)_2]$[7]	532	---	---	1.12	RSA	[S14]
Zn_2(TPyP)(Zn/Cu)/PDMS[8]	532	---	---	4.65×10^{-3}	RSA	[S14]
Zn_2(TPyP)(Zn/Mn)/PDMS	532	---	---	3.1×10^{-3}	RSA	[S14]
Zn_2(TPyP)(Zn/Zn)/PDMS	532	---	---	1.35×10^{-3}	RSA	[S14]
Zn_3(TPyP)/PDMS	532	---	---	4×10^{-4}	RSA	[S14]
TPyP(Cu) /PDMS	532	---	---	4×10^{-5}	RSA	[S14]
TPyP(Zn)/PDMS	532	---	---	2×10^{-4}	RSA	[S14]
TPyP(Mn)/PDMS	532	---	---	1.6×10^{-4}	RSA	[S14]
TPyP(Ni)/PDMS	532	---	---	7.24×10^{-5}	RSA	[S14]
ZnTPyP(Cu)[9]	532	9.2×10^{-1}	Self-defoc.	5.7×10^{-1}	RSA	[S15]
ZnTPyP(Mn)	532	6.6×10^{-1}	Self-defoc.	1.2×10^{-1}	RSA	[S15]
ZnTPyP(Ni)	532	7.1×10^{-1}	Self-defoc.	1.8×10^{-1}	RSA	[S15]
ZnTPyP(H_2)	532	6.5×10^{-2}	Self-defoc.	5.1×10^{-2}	RSA	[S15]
Zn-TCPP/UiO66[10]	532	10.5×10^{-5}	Self-foc.	10.3×10^{-5}	RSA	[S16]
Zn-MOF[11]	532	6×10^{-4}	Self-foc.	6.8×10^{-4}	RSA	[S17]
ZnCu-MOF[12]	532	8.5×10^{-4}	Self-defoc	2.15×10^{-3}	RSA	[S17]
ZnCu-MOF-1	532	2×10^{-3}	Self-defoc	1.15×10^{-3}	RSA	[S17]
Ni-Zn-MOF NSs[13]	532	---	---	1.24×10^{-6}	RSA	[S18]
Ni-Co-MOF NSs[13]	532	---	---	1.12×10^{-5}	RSA	[S18]
Ni-Cd-MOF NSs[13]	532	---	---	1.28×10^{-5}	RSA	[S18]
Cd-CA[14]	530	2.33×10^{-7}	Self-foc.	14.13×10^{-8}	TPA	[S19]
Cd-CA[14]	415	1.65×10^{-7}	Self-foc.	11.98×10^{-8}	TPA	[S19]
BSA-CuA	550	1.44×10^{-8}	Self-foc.	6.62×10^{-8}	TPA	[S20]
BSA-CuCl	550	1.12×10^{-8}	Self-foc.	5.40×10^{-8}	TPA	[S20]
$CsPbBr_3$	1000	---	---	5×10^{-6}	TPA	[S21]
$CsPbBr_3$ Nanoplate	800	---	---	3.9×10^{-6}	TPA	[S22]
$CH_3NH_3PbBr_3$	800	---	---	8.6×10^{-6}	TPA	[S23]
$CsPbBr_3$	800	3.52×10^{-4}	Self-foc.	3.9×10^{-11}	TPA	[S24]
Ag–MoS_2–rGO	532	---	---	9.2×10^{-5}	TPA	[S25]
Nh_2-Zn-MUM-6	532	30.83	Self-foc.	12.82	TPA	This work

[1] $Cu_6(^tBuS)_2(MBIZ)_4$ (MBIZ = 2-mercaptobenzimidazole; ^{t}BuS = tert-butyl mercaptan)

[2] $Cu_8(^tBuS)_4(MBIZ)_4\cdot CH_3OH\cdot 2H_2O$ (MBIZ = 2-mercaptobenzimidazole; ^{t}BuS = tert-butyl mercaptan)

[3] Porphyrin-based surface-supported metal–organic framework nanofilms

[4] $[Co(BH(2\text{-}mim)_3)(BTC)_{1/3}](HBH(2\text{-}mim)_3)_{1/3}(NMA)$ (2-mim=2-methylimidazole and NMA=N-methylacetamide), 1,3,5-benzenetricarboxylate (BTC)

[5] $[Zn(BH(2\text{-}mim)_3)(CHTC)_{1/3}]$ (2-mim = 2-methylimidazole) and 1,3,5-cyclohexanetricarboxylate (CHTC) ligands

[6] $[Zn_2(TPyP)(AC)_2]$ (TPyP = 5,10,15,20-tetra(4-pyridyl)porphyrin and AC = acetate)

[7] $[Zn_3(TPyP)(H_2O)_2(C_2O_4)_2]$ (TPyP = 5,10,15,20-tetra(4-pyridyl)porphyrin and C_2O_4 = oxalate)

[8](TPyP = 5,10,15,20-tetra(4-pyridyl)porphyrin)

[9](TPyP = 5,10,15,20-tetra(4-pyridyl)porphyrin)

[10](TCPP = tetrakis(4-carboxyphenyl)porphyrin)

[11]Zn-MOF: $\{[ZnL(bpe)_{0.5}]\cdot 0.5(H_2bpe)\cdot 3H_2O\}_n$ (1,2-bis(4-pyridyl)ethylene (bpe))

[12]ZnCu-MOF $\{[Zn_{0.78}Cu_{0.22}L(bpe)_{0.5}]\cdot 0.5(H_2bpe)\cdot 3H_2O\}_n$ (1,2-bis(4-pyridyl)ethylene (bpe))

[13]Ni-M-MOF (M = Zn, Co, Cd) ($[Ni_3(OH)_2(1,4\text{-}BDC)_2\text{-}(H_2O)_4]\cdot 2H_2O$; 1,4-BDC = dimethyl phthalate)

[14] Citraconic acid-directed supramolecular metallogel of Cd(II).

Supporting References

(S1) *CrystalClear-SM Exper*t v2.1. Rigaku Americas, *The Woodlands, Texas, USA*, and Rigaku Corporation, *Tokyo, Japan*, **2015**.

(S2) Sheldrick, G. M. SHELXT – Integrated space-group and crystal structure determination. *Acta Crystallogr., Sect. A: Found. Adv*. **2015**, *71*, 3-8.

(S3) Sheldrick, G. M. Crystal structure refinement with SHELXL. *Acta Crystallogr., Sect. C: Struct. Chem.* **2015**, *71*, 3-8.

(S4) Spek, A. L. PLATON SQUEEZE: a tool for the calculation of the disordered solvent contribution to the calculated structure factors. *Acta Crystallogr. Sect C: Struct. Chem.* **2015**, *71*, 9-18.

(S5) Spek, A. L. Structure validation in chemical crystallography. *Acta Crystallogr. Sect D: Biol. Crystallogr.* **2009**, *65*, 148-155.

(S6) Dolomanov, O. V.; Bourhis, L. J.; Gildea, R. J.; Howard, J. A. K.; Puschmann, H. OLEX2: a complete structure solution, refinement and analysis program. *J. Appl. Crystallogr*. **2009**, *42*, 339-341.

(S7) Blatov, V. A. *IUCr CompComm Newsletter* **2006**, *7*, 4.

(S8) Blatov, V. A.; Shevchenko, A. P.; Proserpio, D. M. Applied topological analysis of crystal structures with the program package ToposPro. *Cryst. Growth Des*. **2014**, *14*, 3576-3586.

(S9) O'Keeffe, M.; Yaghi, O.M. Deconstructing the crystal structures of metal–organic frameworks and related materials into their underlying nets. *Chem. Rev*. **2012**, *112*, 675-702.

(S10) Li, M.; Li, D.; O'Keeffe, M.; Yaghi, O.M. Topological analysis of metal–organic frameworks with polytopic linkers and/or multiple building units and the minimal transitivity principle. *Chem. Rev*. **2014**, *114*, 1343-1370.

(S11) Cheng, Y.J.; Wu, R.R.; Zhao, J.Q.; Chen, E.X.; Zhou, X.; Dai, Y.; Zheng, H.L.; Lin, Q. Ligand Modification on Copper (I)-Sulfide Clusters for Enhanced Third-Order Nonlinear Optical Response. *Cryst. Growth Des.* **2023**, *23*, 5421−5427.

(S12) Gu, C.; Zhang, H.; You, P.; Zhang, Q.; Luo, G.; Shen, Q.; Wang, Z.; Hu, J. Giant and Multistage Nonlinear Optical Response in Porphyrin-Based Surface-Supported Metal–Organic Framework Nanofilms. *Nano Lett.* **2019**, *19*, 9095−9101.

(S13) Hong, Q.L.; Zhao, G.X.; Chen, J.Q.; Chen, S.; Zhang, H.X.; Zhang, J. Fine-Tuning Tridentate Ligands for the Construction of Nanotube-Based Boron Imidazolate Frameworks with High Chemical Stability. *Inorg. Chem.* **2023**, *62*, 618−623.

(S14) Li, D.J.; Li, Q.H.; Wang, Z.R.; Ma, Z.Z.; Gu, Z.G.; Zhang, J. Interpenetrated Metal-Porphyrinic Framework for Enhanced Nonlinear Optical Limiting. *J. Am. Chem. Soc.* **2021**, *143*, 17162-17169.

(S15) Gu, C.; Zhang, H.; You, P.; Zhang, Q.; Luo, G.; Shen, Q.; Wang, Z.; Hu, J. Giant and Multistage Nonlinear Optical Response in Porphyrin-Based Surface-Supported Metal–Organic Framework Nanofilms. *Nano Lett*. **2021**, *21*, 10012–10018.

(S16) Yin, X.; Sun, Y.; Geng, K.; Cui, Y.; Huang, J.; Hou, H. Ingenious Modulation of Third-Order Nonlinear Optical Response of Zr-MOFs through Defect Engineering Based on a Mixed-Linker Strategy. *Inorg. Chem*. **2024**, *63*, 6723–6733.

(S17) Yu, J.; Sun, Y.; Geng, K.; Huang, J.; Cui, Y.; Hou, H. Third-Order Nonlinear Optical Modulation Behavior of Photoresponsive Bimetallic MOFs. *Inorg. Chem*. **2024**, *63*, 6526–6536.

(S18) Zhou, X.; Li, S.; Mao, A.; Wang, Q.; Yang, J.; Zheng, J.; Wen, N.; Zhan, H.; Zheng, Y.Y.; Wei, Y. Novel Binary Ni-Based Mixed Metal–Organic Framework Nanosheets Materials and Their High Optical Power Limiting. *ACS omega* **2022**, *7*, 10429–10437.

(S19) Singha, T.; Pal, B.; Majumdar, S.; Lepcha, G.; Pal, I.; Ray, P.P.; Dey, B.; Datta, P.K. Optical Nonlinearity of Semiconducting Cd (II) Metallogel in the Femtosecond Regime with Two-, Three-, and Four-Photon Absorption. *ACS Appl. Optic. Mater.* **2024**, *2*, 474-484.

(S20) Majumdar, S.; Singha, T.; Dhibar, S.; Mandal, A.; Datta, P.K.; Dey, B. Protein-Based Self-Healing Cu (II)-Metallohydrogels: Efficient Third-Order Nonlinear Optical Materials in Terms of an Intensity-Dependent Refractive Index and Two-Photon Absorption. *ACS Appl. Electron. Mater.* **2020**, *2*, 3678-3685.

(S21) Saouma, F.O.; Stoumpos, C.C.; Kanatzidis, M.G.; Kim, Y.S.; Jang, J.I. Multiphoton Absorption Order of $CsPbBr_3$ as Determined by Wavelength-Dependent Nonlinear Optical Spectroscopy. *J. Phys. Chem. Lett.* **2017**, *8*, 4912–4917.

(S22) Ketavath, R.; Katturi, N. K.; Ghugal, S. G.; Kolli, H. K.; Swetha, T.; Soma, V. R.; Murali, B. Deciphering the Ultrafast Nonlinear Optical Properties and Dynamics of Pristine and Ni-Doped $CsPbBr_3$ Colloidal Two-Dimensional Nanocrystals. *J. Phys. Chem. Lett.* **2019**, *10*, 5577– 5584.

(S23) Walters, G.; Sutherland, B.R.; Hoogland, S.; Shi, D.; Comin, R.; Sellan, D.P.; Bakr, O.M.; Sargent, E.H. Two-Photon Absorption in Organometallic Bromide Perovskites. *ACS Nano* **2015**, *9*, 9340–9346.

(S24) Ketavath, R.; Katturi, N.K.; Ghugal, S.G.; Kolli, H.K.; Swetha, T.; Soma, V.R.; Murali, B. Deciphering the Ultrafast Nonlinear Optical Properties and Dynamics of Pristine and Ni-doped $CsPbBr_3$ Colloidal Two-Dimensional Nanocrystals. *J. Physic.Chem. Lett.* **2019**, *10*, 5577-5584.

(S25) TC, S.G. Genuine Two-Photon Absorption and Optical Limiting Property of the Ag–rGO–MoS_2 Hybrid: Implications for Laser Safety Devices. *ACS Appl. Nano Mater.* **2024**, *7*, 3885-3896.
